# Growth differentiation factor‐15 is associated with muscle mass in chronic obstructive pulmonary disease and promotes muscle wasting *in vivo*

**DOI:** 10.1002/jcsm.12096

**Published:** 2015-12-29

**Authors:** Mehul S. Patel, Jen Lee, Manuel Baz, Claire E. Wells, Susannah Bloch, Amy Lewis, Anna V. Donaldson, Benjamin E. Garfield, Nicholas S. Hopkinson, Amanda Natanek, William D‐C Man, Dominic J. Wells, Emma H. Baker, Michael I. Polkey, Paul R. Kemp

**Affiliations:** ^1^ NIHR Respiratory Biomedical Research UnitRoyal Brompton & Harefield NHS Foundation Trust and Imperial CollegeLondonUK; ^2^ Section of Molecular MedicineNational Heart and Lung Institute, Imperial College LondonLondonUK; ^3^ Institute of Infection and ImmunitySt George's, University of LondonLondonUK; ^4^ Comparative Biomedical Sciences Royal Veterinary CollegeLondonUK

**Keywords:** Atrophy, GDF‐15, Muscle mass, COPD, Electroporation

## Abstract

**Background:**

Loss of muscle mass is a co‐morbidity common to a range of chronic diseases including chronic obstructive pulmonary disease (COPD). Several systemic features of COPD including increased inflammatory signalling, oxidative stress, and hypoxia are known to increase the expression of growth differentiation factor‐15 (GDF‐15), a protein associated with muscle wasting in other diseases. We therefore hypothesized that GDF‐15 may contribute to muscle wasting in COPD.

**Methods:**

We determined the expression of GDF‐15 in the serum and muscle of patients with COPD and analysed the association of GDF‐15 expression with muscle mass and exercise performance. To determine whether GDF‐15 had a direct effect on muscle, we also determined the effect of increased GDF‐15 expression on the *tibialis anterior* of mice by electroporation.

**Results:**

Growth differentiation factor‐15 was increased in the circulation and muscle of COPD patients compared with controls. Circulating GDF‐15 was inversely correlated with *rectus femoris* cross‐sectional area (*P* < 0.001) and exercise capacity (*P* < 0.001) in two separate cohorts of patients but was not associated with body mass index. GDF‐15 levels were associated with 8‐oxo‐dG in the circulation of patients consistent with a role for oxidative stress in the production of this protein. Local over‐expression of GDF‐15 in mice caused wasting of the *tibialis anterior* muscle that expressed it but not in the contralateral muscle suggesting a direct effect of GDF‐15 on muscle mass (*P* < 0.001).

**Conclusions:**

Together, the data suggest that GDF‐15 contributes to the loss of muscle mass in COPD.

## Introduction

Many patients with chronic obstructive pulmonary disease (COPD) find that common activities of daily living become increasingly difficult as they fatigue easily and lose strength.^1^ This reduced functional capacity is a consequence of a reduction in the quantity of muscle and the oxidative capacity of the muscle. In addition to an impaired quality of life, both the reduction in strength and oxidative capacity are associated with an increase in mortality.^2^, ^3^ Consequently, the mechanisms and factors regulating muscle mass are of significant interest.

Growth differentiation factor‐15 (GDF‐15) is a stress responsive growth regulator that is elevated in a number of diseases including heart failure,^4^ pulmonary arterial hypertension,^5^, ^6^ and diabetes^7^ as well as in patients on the intensive care unit.^8^ This member of the transforming growth factor‐β (TGF‐β) family is associated with mortality in heart failure,^4^ and recent studies demonstrate that serum GDF‐15 levels are a predictor of all‐cause mortality in elderly individuals.^9^, ^10^ The mechanism linking the protein to mortality is not clear, but some evidence suggests that GDF‐15 may promote muscle wasting perhaps providing such a link. For example, we showed that in patients admitted to the intensive care unit following heart surgery, plasma GDF‐15 levels remained elevated in patients who showed significant muscle wasting 7 days after surgery but not in those whose muscle mass did not fall.^11^ Importantly, we also found that myotubes treated with GDF‐15 showed a reduction in diameter confirming atrophy. Furthermore, muscle wasting in mice implanted with GDF‐15‐expressing tumours was prevented by treating the mice with an anti‐GDF‐15 antibody.^12^ In this latter study, pair feeding experiments suggested that this muscle wasting resulted from a suppression of appetite—a feature common in the later stages of chronic diseases including COPD.

The biological stresses that can elevate GDF‐15 include hypoxia,^13^ oxidative stress,^14^ and increased inflammatory cytokines,^15^ all features relevant in the pathogenesis of COPD. We hypothesized that patients with COPD would have elevated GDF‐15 and that the levels of this growth factor would be inversely associated with muscle mass. We also hypothesized that the effects of increased GDF‐15 on muscle mass would be both directly on the muscle and indirectly through appetite suppression.

## Methods

### Study participants

Ethical approval was obtained, and all participants provided written informed consent prior to study testing within the Royal Brompton & Harefield NHS Foundation Trust (original cohort and controls; ‘RBH cohort’) or at St George's Healthcare NHS Trust (‘SGH cohort’ REC 10/H0721/75). Stable COPD patients were diagnosed according to Global Initiative in Chronic Obstructive Lung Disease (GOLD) guidelines^16^ and recruited from outpatient clinics. Controls were recruited via an ethically approved database (REC H/H1102/36). Exclusion criteria for all participants included a physician diagnosis of heart, renal or liver failure, significant neurological or musculoskeletal limitation to mobility, and for patients, exacerbation within the past 4 weeks. Specimens from the RBH cohort and controls were previously used for our investigation of the role of Klotho.^17^

Measurements included spirometry—we report the FEV_1_ expressed as per cent predicted (FEV_1_%pred),^18^ plethysmographic lung volumes (RV/TLC),^19^, ^20^ carbon monoxide diffusing capacity (K_CO_) (CompactLab system; Jaeger, Wurzburg, Germany),^19^ modified medical research council dyspnoea score (MMRC),^21^ six minute walk distance (6 MW) or incremental shuttle walk distance (ISW),^22^ health status (St George's Respiratory Questionnaire (SGRQ) or COPD assessment test (CAT)^23^), fat free mass and fat free mass index (FFMI),^24^ dominant leg quadriceps strength expressed normalized to body mass index (QMVC/BMI),^25^ *rectus femoris* cross‐sectional area (RF_CSA_), and daily step count.^26^ The 6 MW, RV/TLC, K_CO_, and SGRQ data were only available in the RBH cohort, while ISW and CAT data were only available in the SGH cohort.

### Sample collection

All participants underwent venesection prior to other testing. A *vastus lateralis* percutaneous needle biopsy was performed as previously described.^27^ All biological samples were stored at −80°C prior to being analysed as a batch.

### Molecular analysis

Serum GDF‐15 [QUANTIKINE © (R&D Systems Inc. Minneapolis, MN, USA)] and 8‐oxo‐dG (Trevigen, Gaithersburg, MD, USA) levels were measured by enzyme‐linked immunosorbent assay. Serum 8‐oxo‐dG measurements were limited to the RBH cohort. Muscle was homogenized, and cDNA was derived as described previously.^27^ Human and mouse muscle mRNA expression was determined by real‐time quantitative PCR (qPCR) as previously described^28^ using the primers described in the Supporting Information (*Table* E2) and normalizing the data to RPLPO. Analysis of data from the RBH cohort was not performed blinded, analysis of the data from the SGH data was blinded as assays were performed at Imperial by SAB, and data were analysed at SGH by EHB.

### Cloning

Full‐length murine GDF‐15 cDNA was sub‐cloned into pGEMT by PCR from an IMAGE clone (Source Bioscience, Nottingham, UK) sequenced then shuttled into pCAGGS to generate pC‐GDF‐15. The pC‐GDF‐15 and empty pCAGGs were amplified using JM109 bacteria and purified using EndoFree Mega kit (Qiagen, Hilden, Germany) according to manufacturer's instructions. Plasmids were eluted in sterile dH_2_O.

### Animal experiments

Mouse experiments were approved by the Royal Veterinary College Ethical Review Process (ERP‐A‐2010‐WS01) and were licenced by the UK Secretary of State for the Home Office as Project License PPL 70/6797. Sample size was estimated from previous experiments as the minimum likely to yield a significant result. The animals used were male C57Bl/6 mice (24.9 ± 0.8 g) that were 8 weeks of age at the start of the experiment. Experiments were performed in two blocks of eight mice generating four control and four experimental animals each time to give a total of eight in each group. Animals were randomly assigned to control or experimental groups. No animal samples were excluded. The analyses were not performed blinded, but all samples were analysed simultaneously for each experimental variable with the exception of animal and muscle weights, which were performed in two blocks as described previously.

### Electroporation

Twenty‐five microlitres of bovine hyaluronidase (0.04U/μL, H‐4272, Sigma, Poole, Dorset, UK) was injected into each *tibialis anterior* muscle of anesthetized mice as previously described.^29^ Mice were left to recover for 2 h at 37 C and then anesthetized with 2% isofluorane, and 25 µg of plasmid (pCAGGS empty or pCAGGS‐GDF‐15) at a concentration of 1 µg/μL was injected into the right *tibialis anterior*, and 25 µg of CAGGS empty was injected into the left *tibialis anterior*. Then electrodes coated in electro‐conductive cream were placed firmly around the *tibialis anterior*, and a voltage of 175 V/cm was applied in ten 20 ms square‐wave pulses at a frequency of 1 Hz using a BTX ECM 830 electroporator (BTX Harvard Apparatus, Holliston, MA, USA).^30^ After electroporation, the mice were left to recover at 37°C before being returned to their normal housing. Mice were fed with normal chow and given water *ad libitum* for 2 weeks, after which the mice were sacrificed by cervical dislocation. *Tibialis anterior* muscles were harvested and placed upright on to small pieces of cork with a small amount of Optimal Cutting Temperature Compound at the bottom to fix the bottom of the *tibiales anteriores* onto cork and snap frozen in liquid nitrogen‐cooled isopentane.

### Sectioning

*Tibialis anterior* muscles were sectioned vertically from the top towards the cork at the bottom, and 10‐µm‐thick sections grouped into 12 evenly spaced levels were cut.^30^ Representative sections from each level were captured onto frost‐free glass slides. The subsequent 30 sections from each level were then collected into micro‐centrifuge tubes and stored at −80°C for mRNA analysis. The slides were allowed to air dry for 30 min before storage at −80°C until use. Muscle sections were stained with haematoxylin and eosin, and random fields were captured at ×20 magnification using an Olympus CKX41 camera and Cell^D software (Olympus Europe, Hamburg, Germany).

### Immunohistochemistry

Sections of *tibialis anterior* were fixed with methanol at −20°C and then washed with phosphate‐buffered saline two times. They were then incubated in 0.3% H_2_O_2_ for 20 min and blocked in goat serum before being incubated overnight with rabbit anti‐GDF‐15 (biorbyt, orb49016, 1:100) at 4 C. Following washing with phosphate‐buffered saline, the sections were incubated with biotinylated anti‐rabbit IgG (Vectastain, ZA0520, 1:200) for 30 min. Sections were washed and then incubated with avidin and biotinylated enzyme mix (Vectastain, Elite ABC reagent PK‐6100 series), underwent further washing, and were then stained with diaminobenzidine. Slides were mounted and imaged at ×20 magnification.

### Luciferase reporter assays

Cells were cultured as described in Martin *et al*.^31^ For assays, 2.5 × 10^4^ cells were seeded per well and incubated overnight. The following day, cells were transfected using lipofectamine as previously described^32^ using 0.4 µg plasmid DNA per well consisting of 0.2 µg of (CAGA)12‐luciferase plasmid, 0.1 µg of pRLTK plasmid 0.1 µg pcDNA3 in 20 μL Opti‐Mem (Invitrogen, Carlsbad, CA, USA). The DNA was added to 20 μL Opti‐MEM containing 2 μL lipofectamine and incubated for 15 min and then diluted in serum‐free DMEM, and 200 μL of this mix was added to each well. After 4.5 h, the media was replaced with DMEM supplemented with 10% (v/v) foetal bovine serum. Twenty‐four hours later, cells were serum starved for 7 h and then washed and treated with serum‐free DMEM containing either GDF‐15 (R&D Systems) or TGF‐β1 for 16 h. Luciferase activity was assayed using the Dual‐Luciferase® Reporter Assay System (Promega, Madison, WI, USA) according to the manufacturer's instructions.

### Statistical analysis

Statistical analyses and graphical presentations were performed using Aabel, GraphPad Prism 5 (GraphPad Software, San Diego, CA, USA) or SPSS version 18 (IBM, Armonk, NY, USA). Significance was set at a two‐tailed *P*‐value of ≤0.05. qPCR data were log transformed; GDF‐8 and GDF‐15 mRNA expressions were normalized to RPLPO expression, except where GDF‐8 and GDF‐15 were compared directly. Human data were analysed by unpaired *t*‐tests (normally distributed data), Mann–Whitney (non‐parametrically distributed data), or χ^2^ tests to compare two groups. Analysis of variance or Kruskal–Wallis, with post‐hoc correction, was used to compare three groups. Normality was determined by Shapiro–Wilk's test. Association between variables was evaluated by univariate and multivariate regression analysis. Results are expressed as mean (standard deviation) or median (interquartile range). Correlation analysis was performed using Pearson's test to look for linear relationships in the data. Mouse data were analysed by paired *t*‐test to analyse the effect of GDF‐15 within animals or unpaired *t*‐test to determine differences between groups. *P*‐values for Mann–Whitney tests and *t*‐tests are quoted to three significant figures, as Aabel does not give accurate *P*‐values for *P* < 0.001.

## Results

### Physiological parameters

Consistent with a diagnosis of COPD, patients had a reduced FEV_1_ and K_CO_, and a significantly increased RV/TLC, MMRC, and SGRQ compared with controls. RF_CSA_ data were available for 12 controls and 38 patients (missing values were a result of the unavailability of equipment during the patient visit) and showed that the quadriceps bulk was smaller in the patients compared with the controls (*Figure* E1). RF_CSA_ correlated with MVC in the cohort (*Figure* E2). The patients had poorer exercise performance marked by 6 MW distance compared with controls (*Table* 1). Fibre‐type proportions were available from 43 COPD patients and 10 controls and showed that the proportion of type II fibres was increased in COPD patients compared with controls (COPD 65 ± 14% type II vs. control 41 ± 17%, *P* < 0.001). Consistent with previous studies,^33^ 6 MW distance was associated with RF_CSA_ (*r* = 0.595, *P* < 0.001), lung function (FEV_1_, *r* = 0.750, *P* < 0.001),^34^ and proportion of type II fibres in the quadriceps (*r* = −0.559, *P* < 0.001).^35^ All of these components were retained as being significantly associated with 6 MW distance in a multivariate analysis.

**Table 1:** Clinical and demographic data in the different cohorts

	Healthy (*n* = 25)	RBH COPD (*n* = 50)	SGH COPD (*n* = 44)	*P*‐value
Age	64 (7)	65 (10)	71 (8)**	<0.001
Gender (male : female)	16:9	28:22	28:16	0.26
Height (m)	1.70 (0.09)	1.64 (0.23)	1.69 (0.08)	0.39
Smoking pack years	0.0 (0, 0)	40 (28, 50)***	50 (36, 67)***	<0.001
FEV_1_ (%pred)	106 (13)	53 (26)***	56 (20)	Not tested
K_CO_ (%pred)	94 (14)	68 (24)**	—	0.001
RV/TLC (%)	40 (17)	50 (12)	—	0.001
BMI (kg/m^2^)	24.9 (4.4)	26.6 (6.9)	24.4 (4.5)	0.07
FFMI (kg/m^2^)	18.2 (3.3)	17.6 (2.8)	16.7 (2.2)	0.11
QMVC/BMI	1.38 (0.3)	1.21 (0.5)	1.22 (0.49)	0.31
MMRC	0.0 (0, 0)	2.0 (1.1)***	1.8 (1.1)***	<0.001
SGRQ	2 (2)	46 (24)***	—	<0.001
CAT	—	—	19.5 (7.0)	—
6 MWT (m)	606 (70)	392 (107)***	—	<0.001
ISWT (m)	—	—	263 (174)	—
Steps per day	10 775 (3881)	4921 (3542)***	3172 (2067)***	<0.001

BMI, body mass index; COPD, chronic obstructive pulmonary disease; IQR, interquartile range; RBH, Royal Brompton Hospital; SD, standard deviation; SGH, St George's Hospital.

Data are expressed as mean (SD) or median (IQR). Differences between groups were assessed by chi‐square and ANOVA or Kruskal–Wallis with post‐hoc analyses by Newman–Keuls or Dunn's multiple comparison test.

* Significant difference of *P* < 0.05 as compared with healthy controls.

** Significant difference of *P* < 0.01 as compared with healthy controls.

*** Significant difference of *P* < 0.001 as compared with healthy controls.

**Figure 1:**
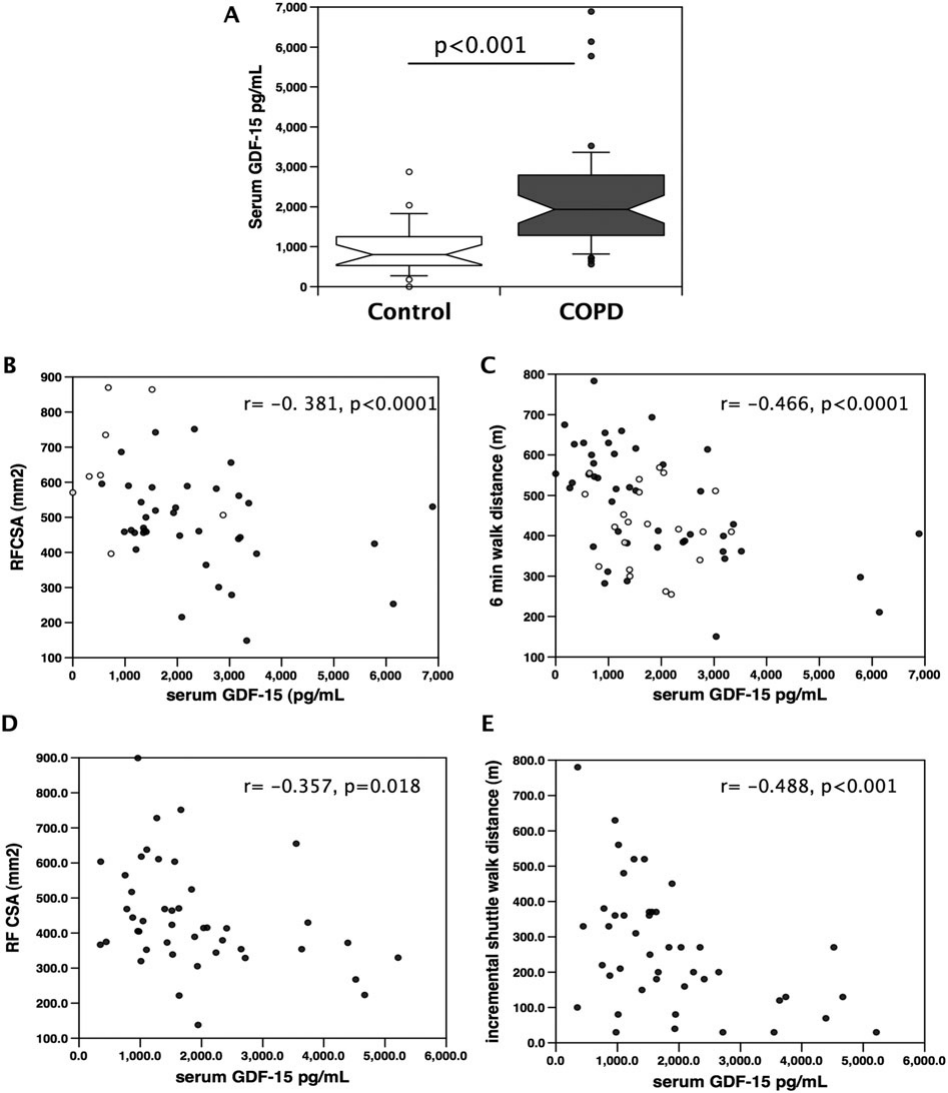
Serum GDF‐15 is elevated in patients with COPD and is associated with muscle size and exercise capacity. Serum levels of GDF‐15 were determined in patients with COPD and age‐matched controls and compared with RF_CSA_ and exercise performance in two cohorts of COPD patients. (A) Serum GDF‐15 was elevated in COPD patients (*n* = 46) compared with healthy controls (*n* = 21, Mann–Whitney, *P* < 0.001). (B, D) RF_CSA_ was inversely correlated (Pearson's *r*) with serum GDF‐15 in two independent cohorts of COPD patients and controls [(B) RBH patients and controls *r* = −0.381, *P* < 0.001; (D) St George's patients only *r* = −0.357, *P* = 0.018]. (C, E) Exercise capacity was inversely correlated with serum GDF‐15 in two independent cohorts of COPD patients and controls [(C) RBH patients and controls measured as 6 MW, *r* = −0.466, *P* < 0.001; (E) St George's patients only, measured as ISW, *r* = −0.488, *P* < 0.001]. Patients are shown as black circles, and controls are shown as open circles. COPD, chronic obstructive pulmonary disease; GDF, growth differentiation factor; RBH, Royal Brompton Hospital.

Patients with COPD from the RBH cohort had a marked elevation of serum GDF‐15 levels compared with healthy controls (*P* < 0.001; *Figure* 1A). In the whole cohort, GDF‐15 expression was associated with RF_CSA_ (*r* = −0. 381, *P* < 0.001; *Figure* 1B) and with exercise capacity (6 MW, *r* = −0.466, *P* < 0.001; *Figure* 1C) but was not associated with BMI or FFMI. In COPD patients alone, the association of GDF‐15 with RF_CSA_ did not reach statistical significance (*r* = −0.298, *P* = 0.073), but the association with 6 MW was retained (*r* = −0.297, *P* = 0.048). Serum GDF‐15 was not associated with type II fibre proportion.

To confirm our findings, we analysed serum samples from a second cohort of COPD patients attending clinics at St George's hospital. These patients had similar lung function, BMI, FFMI, and strength to the RBH cohort but were older (*P* < 0.001) and took fewer steps per day (*P* = 0.021). Analysis of these patients showed a similar association of serum GDF‐15 with RF_CSA_ (*r* = −0.357, *P* = 0.018; *Figure* 1D) and was also associated with exercise capacity analysed by incremental shuttle walk distance (ISW, *r* = −0.488, *P* < 0.001; *Figure* 1E).

To further analyse the associations of GDF‐15 with clinical parameters, the patient data from the RBH and SGH cohorts were combined, and univariate and multivariate regression analysis was performed. Male gender and age were associated with higher GDF‐15 levels, while RF_CSA_ QMVC/BMI, ISW, and daily steps were negatively associated with GDF‐15. In the multivariate analysis, age, male gender, and RF_CSA_ were found to have independent associations with serum GDF‐15 (*Table* 2).

**Table 2:** The univariate and multivariate relationships between serum GDF‐15 levels and clinical parameters in COPD patients in the RBH and SGH cohorts

		Univariate regression			Multiple regression		
Parameter	Variable	Coefficient (95% CI)	Standardized coefficient	*P*	Coefficient (95% CI)	Standardized coefficient	*P*
Demographics	Age	58 (32, 84)	0.42	<0.001	52 (18, 88)	0.33	0.004
	Gender (male)	619 (79, 1160)	0.23	0.03	818 (209, 1428)	0.30	0.009
	Current smoker	−644 (−1199, −90)	−0.24	0.02	−276 (−863, 311)	−0.10	0.35
	Smoking pack years	5 (−4, 14)	0.12	0.25	—	—	—
Lung function	FEV_1_ (%pred)	1 (−10, 13)	0.02	0.82	—	—	—
	K_CO_ (%pred)	14 (−5, 32)	0.23	0.14	—	—	—
	RV/TLC (%)	−25 (−61, 12)	−0.20	0.18	—	—	—
Muscle parameters	BMI	25 (−21, 71)	0.12	0.27	—	—	—
	FFMI	101 (−4, 205)	0.20	0.06	—	—	—
	QMVC/BMI	−617 (−1185, ‐51)	−0.22	0.03	−303 (−1011, 406)	−0.10	0.40
	RF_CSA_	−2.7 (−4.7, −0.7)	−0.28	0.01	−2.4 (−4.6, −0.3)	−0.25	0.03
Dyspnoea/health status	MMRC	212 (−33, 457)	0.18	0.09	—	—	—
	SGRQ (RBH only)	4 (−14, 22)	0.07	0.66	—	—	—
	CAT (SGH only)	43 (−9, 95)	0.25	0.10	—	—	—
Exercise capacity/physical activity	6 MW (RBH only)	−2 (−6, 2)	‐0.15	0.30	—	—	—
	ISW (SGH only)	−3 (−5, −2)	−0.49	0.001	Not included in multivariate analysis as data not available only available in 44 patients		
	Steps per day	−0.10 (−0.19, −0.01)	−0.23	0.03	0.03 (−0.07, 0.13)	0.07	0.51

BMI, body mass index; CI, confidence interval; COPD, chronic obstructive pulmonary disease; GDF, growth differentiation factor; RBH, Royal Brompton Hospital; SGH, St George's Hospital.

**Figure 2:**
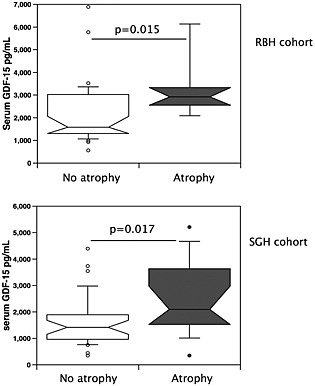
GDF‐15 is elevated in COPD patients with muscle atrophy. Serum GDF‐15 levels were compared in patients defined as having muscle atrophy (as described in the Methods section, *n* = 20) and those with a normal muscle size (*n* = 69) for both the RBH and St George's cohorts. Patients with atrophy had higher serum GDF‐15 levels than those without atrophy in both cohorts (RBH, *P* = 0.011, and St George's, *P* = 0.022, Mann–Whitney test). COPD, chronic obstructive pulmonary disease; GDF, growth differentiation factor; RBH, Royal Brompton Hospital; SGH, St George's Hospital.

We have previously described normal RF_CSA_ values in 40 healthy individuals with a similar demographic to the present cohort (640 (136) mm^2^ 26). Using this value, we defined *rectus femoris* atrophy as an RF_CSA_ measurement two standard deviations below the mean (<368 mm^2^). In both cohorts, patients with atrophy had higher serum GDF‐15 than patients without atrophy (*Figure* 2).

### Circulating growth differentiation factor‐15 levels relate to levels of oxidative stress

Circulating GDF‐15 levels are associated with oxidative stress marked by 8‐oxo‐dG in patients with renal failure. We therefore determined whether serum 8‐oxo‐dG was increased in COPD patients and if it associated with GDF‐15. Serum 8‐oxo‐dG was not different between controls and COPD patients (*P* = 0.38; *Figure* E3). Considering all participants in whom 8‐oxo‐dG measurements were made (*n* = 75), there was only a significant univariate relationship between 8‐oxo‐dG levels and BMI (standardized *β* 0.32, *P* = 0.005); although there were trends towards an association between 8‐oxo‐dG levels with FFMI (*P* = 0.06), RV/TLC (*P* = 0.08), and serum GDF‐15 (*P* = 0.06), these relationships did not quite reach statistical significance. When limiting the analysis to COPD patients (*Table* E1), only BMI (standardized *β* 0.44; *P* = 0.002), FFMI (standardized *β* 0.37; *P* = 0.009), and serum GDF‐15 (standardized *β* 0.31; *P* = 0.03) were associated with 8‐oxo‐dG levels, although none of these relationships were retained in a multivariate analysis.

### Increased growth differentiation factor‐15 expression is observed in the quadriceps of chronic obstructive pulmonary disease patients

**Figure 3:**
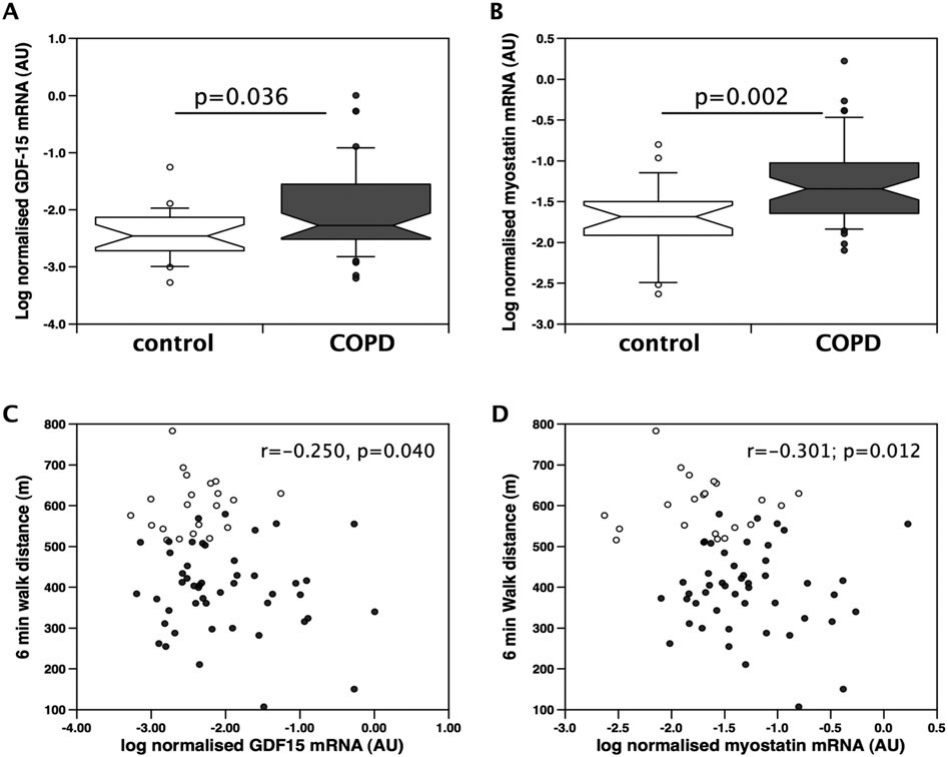
Expression of GDF‐15 and myostatin is elevated in the muscle of patients with COPD. The expression of (A) GDF‐15 and (B) myostatin was determined by real‐time quantitative PCR in skeletal muscle biopsies of patients (*n* = 49) and controls (*n* = 21) from the Royal Brompton Hospital cohort as described in the Methods section. The expression of both mRNAs was higher in the patients than in the controls (GDF‐15, *P* < 0.001; myostatin, *P* < 0.001 Mann–Whitney). (C) Muscle GDF‐15 expression and (D) myostatin expression were also weakly correlated with exercise capacity measured as 6 MW in this cohort (*r* = 0.250, *P* = 0.04, and *r* = 0.301, *P* = 0.012, respectively, Pearson's *r*). COPD, chronic obstructive pulmonary disease; GDF, growth differentiation factor.

Quadriceps muscle biopsies, with sufficient tissue to evaluate GDF‐15 expression, were available from 22 healthy controls and 49 COPD patients from the RBH cohort. GDF‐15 mRNA expression was higher in quadriceps biopsies from COPD patients than from healthy controls (*P* = 0.036, *Figure* 3A). As GDF‐8 (myostatin) is a well‐known regulator of muscle mass in disease, we determined the expression of this TGF‐β ligand in the muscle samples. Quadriceps GDF‐8 mRNA expression was also elevated in COPD patients as compared with healthy controls (*P* = 0.002, *Figure* 3B). Quadriceps GDF‐8 and GDF‐15 mRNA showed a strong linear correlation, *r* = 0.59; *P* < 0.001 (*Figure* E4). There was no relationship between serum GDF‐15 concentrations and muscle GDF‐15 mRNA expression (*r* = 0.10, *P* = 0.35). Both GDF‐15 and GDF‐8 mRNA were inversely correlated with 6 MW distance (*r* = −0.250, *P* = 0.040, and *r* = −0.301, *P* = 0.012, respectively, *Figure* 3C and 3D). The univariate associations with quadriceps GDF‐15 mRNA expression in COPD patients considered alone are presented in *Table* 3. GDF‐15 expression was not associated with RF_CSA_, FFMI, and the proportion of type II fibres in the muscle. This latter observation suggests either that the GDF‐15 expression in the muscle is not limited to the type II fibres or that significant inter‐individual variation in GDF‐15 expression obscures association with fibre type.

**Table 3:** The univariate and multivariate relationships with quadriceps GDF‐15 expression and other clinical parameters in COPD patients

		Univariate regression		
Parameter	Variable	Coefficient (95% CI)	Standardized coefficient	*P*
Demographics	Age	−0.02 (−0.04, 0.01)	−0.27	0.06
	Gender (male)	−0.05 (−0.50, 0.40)	−0.04	0.81
	Current smoker	0.46 (0.03, 0.89)	0.30	0.04
	Pack years	−0.003 (−0.012, 0.006)	−0.09	0.52
Lung function	FEV_1_ (%pred)	−0.003 (−0.012, 0.005)	−0.11	0.44
	K_CO_ (%pred)	−0.007 (−0.014, 0.001)	−0.25	0.09
	RV/TLC (%)	0.01 (−0.01, 0.03)	0.15	0.31
Muscle parameters	BMI	−0.00 (−0.04, 0.03)	−0.03	0.83
	FFMI	−0.02 (−0.09, 0.06)	−0.06	0.69
	QMVC/BMI	−0.07 (−0.54, 0.40)	−0.04	0.77
	RF_CSA_	−0.001 (−0.002, 0.001)	−0.13	0.43
Dyspnoea/health status	MMRC	0.03 (−0.16, 0.22)	0.05	0.72
	Health status (SGRQ)	0.00 (−0.01, 0.02)	0.13	0.38
Exercise capacity/physical activity	Exercise (6 MW)	0.00 (0.00, 0.00)	−0.11	0.44
	Steps per day	0.00 (0.00, 0.00)	0.15	0.33

BMI, body mass index; CI, confidence interval; COPD, chronic obstructive pulmonary disease; GDF, growth differentiation factor.

### Growth differentiation factor‐15 causes muscle wasting in mice

Previous studies in mice have suggested that the association of GDF‐15 with muscle atrophy is due to a reduction in appetite.^12^ However, GDF‐15 was not associated with BMI in either cohort, raising the possibility that in our patients, there was a direct effect of GDF‐15 on muscle, which contributed to the reduction in muscle mass to a greater extent than appetite suppression. To address this possibility, we electroporated the *tibialis anterior* of a group of mice with an expression vector for GDF‐15 and the contralateral *tibialis anterior* with an empty vector. To account for systemic effects of GDF‐15, we also electroporated both *tibiales anteriores* of a second control set of mice with control vector.

**Figure 4:**
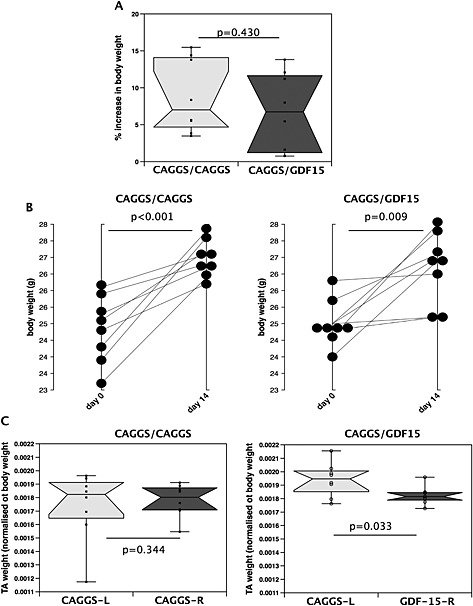
Expression of GDF‐15 in the skeletal muscle of mice does not reduce growth but does cause muscle wasting. Mice were electroporated with either a control vector into both *tibiales anteriores* (pCAGGS1/pCAGGS2, *n* = 8) or a control vector into one *tibialis anterior* and a GDF‐15 expression vector into the contralateral *tibialis anterior* (pCAGGS3/GDF‐15, *n* = 8) and then left for 14 days. (A) There was no difference in the body weight of the mice 14 days after electroporation and (B) no difference in the growth rate of the mice. (C) There was no difference in the proportionate size of the *tibialis anteriores* in control mice (pCAGGS1 and pCAGGS2), but the GDF‐15‐expressing *tibiales anteriores* were smaller than the control *tibiales anteriores* in the experimental animals (pCAGGS3 and GDF‐15 *P* = 0.033 paired *t*‐test). Control *tibiales anteriores* from the experimental mice were not smaller than the *tibiales anteriores* from the control mice, suggesting that there was no systemic effect of GDF‐15. GDF, growth differentiation factor. TA, (*Tibialis anteriores*)

**Figure 5:**
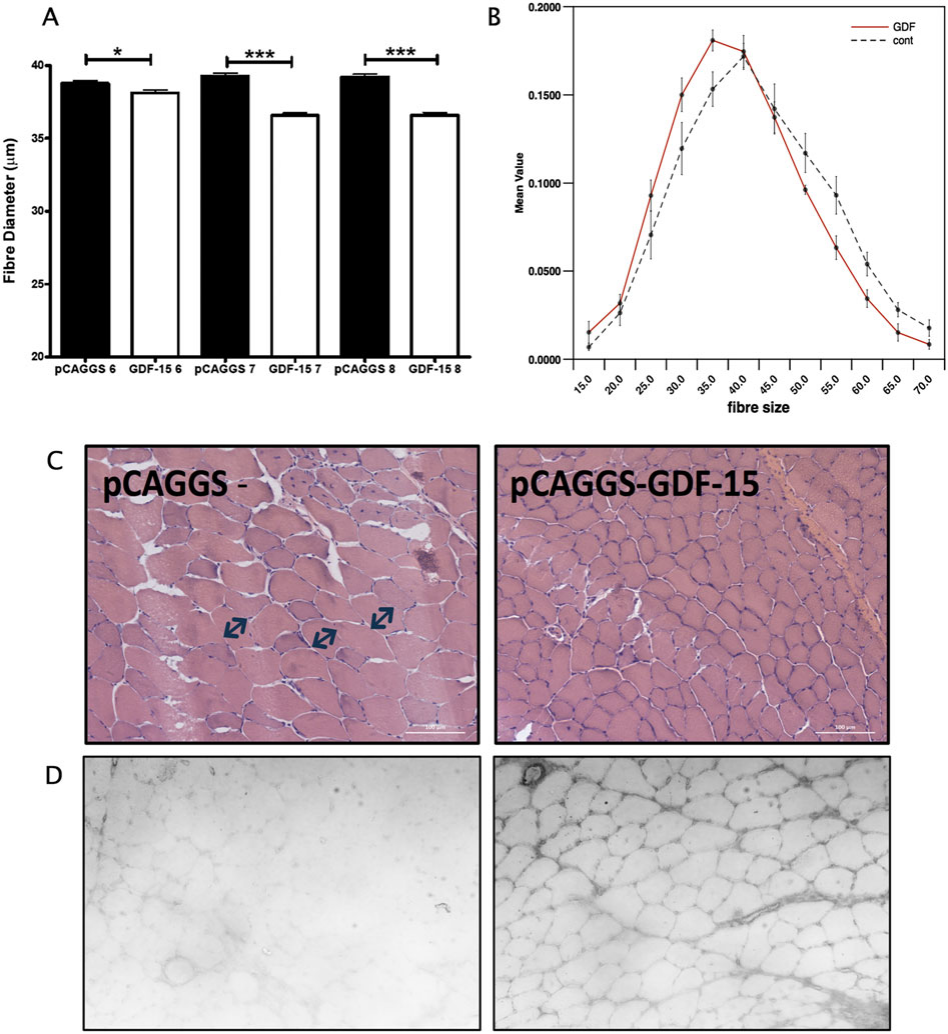
GDF‐15 over‐expression causes muscle fibre atrophy. *Tibialis anteriores* muscles from mice (*n* = 8) over‐expressing GDF‐15 in one *tibialis anterior* were mounted onto cork and then sectioned as described in the Methods section. Myofibre diameter was measured in sections taken from levels 6–8 (representing the middle of the muscle), and minimum feret's diameter was measured. (A) Average fibre diameter was smaller at all three levels of the muscle in the GDF‐15‐expressing *tibialis anterior* (*P* = 0.02 at level 6, *P* < 0.001 at levels 7 and 8, unpaired *t*‐test). (B) Fibres were binned into 12 bins of fibre size ranging from less than 15 µm to more than 65 µm, and the proportion of fibres in each bin was determined. Consistent with the reduction in average fibre diameter, there was a leftward shift in fibre‐type distribution profile of the GDF‐15‐expressing muscle compared with the contralateral muscle. (C) Representative images of control (pCAGGS) and GDF15‐over‐expressing *tibialis anteriors* stained with haemotoxylin and eosin. (D) Representative images of sections from control (pCAGGS, left‐hand image) and GDF‐15 over‐expressing (right‐hand image) stained for GDF‐15. GDF, growth differentiation factor.

The expression of GDF‐15 was significantly increased in the GDF‐15‐electroporated muscle compared with the control‐electroporated muscle indicating successful transfection of the tissue (*Table* 4), and immunostaining of muscle showed increased GDF‐15 protein (*Figure* 5). Protein staining was particularly strong around the cells and in the vessels suggesting that over‐expression led to secretion of the protein. There was no effect of GDF‐15 on final body weight (*Figure* 4A), and mice expressing GDF‐15 in one *tibialis anterior* (experimental mice) increased in weight to the same extent as mice electroporated with control vector in both *tibiales anteriores* (control mice), indicating that there was no effect of expression of GDF‐15 on growth rate (*Figure* 4B). A comparison of the *tibialis anterior* : body weight ratio showed that the size of control‐electroporated *tibiales anteriores* was independent of the expression of GDF‐15 in the contralateral *tibialis anterior* (*Figure* 4C) as the control *tibiales anteriores* from the experimental mice were not smaller than the *tibiales anteriores* from the control mice, suggesting that there was no systemic effect of GDF‐15. However, GDF‐15‐expressing *tibiales anteriores* were smaller than control‐electroporated *tibiales anteriores* from the same mouse (*Figure* 4C). Fibre size was determined at three different levels of the muscle (levels 6–8, see Methods section, which are towards the centre of the muscle) in the GDF‐15‐expressing *tibialis anterior* and the contralateral muscle. GDF‐15 caused a marked reduction in fibre diameter from 39.3 ± 0.2 µm to 36.6 ± 0.2 µm indicating that atrophy or a suppression of regeneration had occurred in the GDF‐15‐expressing muscle and a leftward shift in the distribution of fibres (*Figure* 5). To determine whether GDF‐15 expression had inhibited regeneration, the number of centralized nuclei per fibre was counted, but no difference in this assessment of regeneration was observed (*Figure* E5). Analysis of gene expression failed to show an increase in MuRF‐1 or atrogin‐1 (*Table* 4).

**Table 4:** Normalized mRNA expression in the *tibiales anteriores* of mice with one *tibialis anterior* electroporated with a GDF‐15 expression vector and the contralateral *tibialis anterior* electroporated with a control vector

Gene	cont (log normalized expression, AU)	SEM	GDF‐15 (log normalized expression, AU)	SEM	Paired *t*‐test
GDF‐15	−2.68	0.12	0.62	0.11	0.002
atrogin	−0.42	0.06	−0.54	0.06	0.109
murf	−0.77	0.05	−0.87	0.04	0.151
myhc2a	−0.28	0.17	−0.62	0.12	0.013
myhc2x	2.20	0.06	2.25	0.06	0.699
myhc 2b	1.60	0.07	1.42	0.08	0.144
myhc I	−1.80	0.16	−1.94	0.18	0.484
cyr61	−1.12	0.05	−1.28	0.04	0.049
CTGF	−1.40	0.04	−1.54	0.05	0.086
PAI‐1	−2.29	0.14	−2.32	0.07	0.867

GDF, growth differentiation factor.

### Gene expression in the muscle

To determine whether GDF‐15 expression modified fibre proportion, the muscle sections were stained for all myosin isoforms, and the expression of MHCI, MHCIIa, MHCIIb, and MHCIIx was determined by qPCR. There was a significant reduction in the expression of MHCIIa in the GDF‐15‐over‐expressing muscles, but none of the other MHCs were significantly altered. We did not see any alteration in the expression of MuRF‐1 or atrogin‐1 suggesting that any change in the expression of these genes occurred prior to 14 days. Analysis of TGF‐β responsive genes showed no change in the expression of PAI‐1, a reduction in the expression of Cyr61 and a trend to a reduction in CTGF expression (*Table* 4) suggesting that there was no marked activation of the classical TGF‐β signalling system by GDF‐15. These observations were consistent with a lack of activation of the CAGA_12_ luciferase reporter by GDF‐15 in myoblasts (*Figure* E5).

## Discussion

The data are consistent with the suggestion that GDF‐15 may contribute directly to the loss of muscle mass that occurs in patients with COPD based on three main observations: firstly, that serum levels of GDF‐15 are inversely proportional to RF_CSA_ in patients; secondly, that muscle GDF‐15 expression is elevated in COPD patients; and thirdly, that over‐expression of GDF‐15 leads to a reduction in fibre size in mice. The association of circulating GDF‐15 levels with muscle mass is not unique to COPD as GDF‐15 has been associated with cancer cachexia^36^ in a number of studies, and we have shown that patients who show wasting of the *rectus femoris* following cardiac surgery are exposed to a more sustained elevation of GDF‐15 than those who do not waste.^11^ Furthermore, the link between GDF‐15 and all‐cause mortality may reflect the effects of this growth factor on muscle homeostasis.

Previous studies on GDF‐15 in mice have suggested that the cachectic effects of GDF‐15 are a result of a central effect suppressing appetite.^12^ However, in our previous study, we also found that exposure of myotubes to GDF‐15 causes wasting raising the possibility of a direct effect of GDF‐15 on muscle mass.^11^ The data presented here suggest that suppression of appetite is not the primary cause of the association of GDF‐15 with muscle mass in COPD for two reasons: firstly, in the patients, there was no association of GDF‐15 with BMI, and secondly, because in the mouse model there was no apparent systemic effect of the expression of GDF‐15 in one *tibialis anterior* on the contralateral *tibialis anterior*. The difference in response of the mouse models (implantation of a GDF‐15‐expressing tumour and electroporation of a muscle) may have arisen because the circulating concentration of GDF‐15 produced by over‐expression in the *tibialis anterior* is lower than that produced in the tumour model and therefore insufficient to suppress appetite. In humans, the circulating concentrations of GDF‐15 that we observed in COPD patients were lower than those seen in some studies of colon and metastatic prostate cancer where serum GDF‐15 levels can exceed 100 ng/mL.^37^ These lower levels of GDF‐15 may also account for the apparent lack of association of GDF‐15 with BMI.

In addition to correlating with RF_CSA_, GDF‐15 was also associated with exercise capacity in both patient cohorts. COPD patients show a fibre shift from a predominance of type I fibres to a predominance of type IIA fibres in the quadriceps muscle, and this change in fibre proportion is associated with a marked reduction in exercise capacity.^38^ However, GDF‐15 expression was not associated with fibre type within the muscle nor was there an association of serum GDF‐15 with fibre proportion. These data indicate that an increase in type II fibre proportion is not the main cause for the increase in GDF‐15 expression observed and that any effect of circulating GDF‐15 on fibre proportion is not a significant contributor to the muscle fibre proportions in COPD patients. Consistent with this suggestion, the mouse model did not show an equivalent fibre shift but showed a trend towards a reduction in the expression of all MHCs. However, the fibre profile of the murine *tibialis anterior* is predominantly type IIX and type IIB fibres so that any shift towards a more glycolytic profile may be hard to identify.

The elevation of GDF‐15 in COPD patients is perhaps not surprising. Factors that elevate GDF‐15 expression include oxidative stress,^14^ hypoxia,^13^ and elevated inflammatory cytokines,^15^ all of which occur in COPD. In this study, we show that circulating GDF‐15 is weakly associated with levels of 8‐oxo‐dG, a marker of oxidative stress. However, it is not possible to tell from the data whether this association is causal.

Recently, we have shown that GDF‐15 is elevated in the serum of patients with established critical care‐associated muscle wasting. In that study, we also showed that plasma levels of GDF‐15 were inversely correlated with muscle expression of myomiRs miR‐1, and miR‐499 and that in cultured myotubes, GDF‐15 could suppress the expression of miR‐1.^39^ These miRNAs have important functions in the development and maintenance of skeletal muscle providing a mechanism by which GDF‐15 may regulate muscle phenotype. Interestingly, we have also shown that miR‐1 is suppressed in the muscle of patients with COPD and that quadriceps expression of both miR‐1 and miR‐499 is directly correlated with FFMI.^27^ It is therefore possible that in COPD, GDF‐15 suppresses the expression of these miRNAs to contribute to the reduction in muscle size. We did not see a reduction in the expression of the same miRNAs in the *tibialis anterior* of mice (data not shown). However, electroporation causes a marked stimulation of regeneration, and it is possible that the effects of GDF‐15 on the expression of these miRNAs are masked by this response.

The precise mechanism by which GDF‐15 may contribute to a reduction in muscle mass in COPD patients is not clear. The mouse data suggest that GDF‐15 causes a local effect as we observed reduced muscle size only in the muscle electroporated with the GDF‐15 expression vector. It seems unlikely that GDF‐15 inhibits regeneration as there was no reduction in centralized nuclei in the GDF‐15‐expressing muscles. Furthermore, we have previously shown that GDF‐15 causes a reduction in myotube diameter in cultured cells and increases the expression of the ubiquitin ligases MuRF‐1 and atrogin, suggesting that it can increase atrophy. However, in the mice, we did not see an increase in MuRF or atrogin, possibly because of the timescale of changes or the complexity of the muscle response to electroporation.

#### Critique of the approach

The study presents a cross‐sectional study of patients showing associations between circulating GDF‐15 with RF_CSA_ and exercise capacity, and as such, the data do not show causation. However, the observation that the same associations are found in two independent cohorts and that over‐expression of GDF‐15 in mice leads to a reduction in muscle size suggests that the associations are robust and that GDF‐15 can play a direct causative role in muscle wasting. Nonetheless, a definitive role for GDF‐15 in the muscle wasting of COPD patients will require the generation of a GDF‐15‐neutralizing antibody that could be used to block GDF‐15 function in humans.

## Conclusions

We show that, in patients with COPD, there is an elevation of GDF‐15 that is associated with a reduction in quadriceps mass and with a reduction in exercise capacity. We also show that in mice, over‐expression of GDF‐15 causes local muscle wasting indicating a direct role for this growth factor in the development of muscle wasting. This mechanism is likely to function in tandem with appetite suppression in COPD, but the relative contribution of each mechanism to muscle wasting remains to be established.

## Ethical statements

The authors of this manuscript certify that they comply with the ethical guidelines for authorship and publishing in the Journal of Cachexia, Sarcopenia, and Muscle 2010;1:7–8 (von Haehling S, Morley JE, Coats AJ, and Anker SD).

*Human subjects*: All subjects gave written informed consent; the COPD study protocol was approved by the Royal Brompton & Harefield NHS Trust Research Ethics Committee (REC 10/H1102/36) and by St George's Healthcare NHS Trust Ethics Committee (REC 10/H0721/75).

*Animal studies*: Animal studies were approved by the Royal Veterinary College Ethical Review Process (ERP‐A‐2010‐WS01) and were licenced by the UK Secretary of State for the Home Office as Project License PPL 70/6797.

## Author contributions

P. K. and M. P. proposed the hypothesis for the study. M. P., A. L., J. L., A. D., and B. E. G. performed the miRNA, mRNA DNA, and cell culture analysis supervised by P. K. M. P. collected the RBH human COPD blood samples and muscle biopsies supervised by M. I. P., and C. E. W. collected the SGH human COPD blood samples supervised by E. H. B. J. L. performed the animal experiments and analysis of the mouse samples under the supervision of D. J. W. and P. K. P. K. wrote the first draft of the paper, and all authors contributed to the revision of the draft and the critical analysis of the project.

## Conflict of interest

Paul Kemp has received personal fees from Astellas Pharmaceuticals and research grants from GSK and AstraZeneca Pharmaceuticals. Michael Polkey has received grants and personal fees from Novartis and Astellas and had consultancy fees or payment for research paid to his institution by Biomarin Novartis, AZ, GSK, Boehringer‐Ingelheim, and Lilly. Amanda Natanek has received personal fees from Astellas Pharmaceuticals and research grants from GSK. Mehul Patel was funded by a research grant from AstraZeneca. Jen Lee, Manuel Baz, Claire Wells, Susannah Bloch, Amy Lewis, Anna Donaldson, Benjamin Garfield, Nicholas Hopkinson, William Man, Dominic Wells, and Emma Baker declare that they have no conflicts of interest with the submitted work.

## Supporting information

Supporting info item
