# Supplementary material for: Growth differentiation factor‐15 is associated with muscle mass in chronic obstructive pulmonary disease and promotes muscle wasting in vivo
Source: J Cachexia Sarcopenia Muscle. 2015 Dec 29;7(4):436–48. doi: 10.1002/jcsm.12096 (PMC4864181; doi:10.1002/jcsm.12096)
Supplement: Supplementary file 1 — Supporting info item [file JCSM-7-436-s001.docx]

**DATA SUPPLEMENT**

**TABLE E1.** The univariate and multivariate relationships between serum oxo-8-DG levels and clinical parameters in COPD patients from the Royal Brompton cohort.

| **Parameter** | **Variable** | **Univariate regression** | | | **Univariate regression** | | |
| --- | --- | --- | --- | --- | --- | --- | --- |
|  |  | **Coefficient**  **(95% CI)** | **Standardised coefficient** | **P** | **Coefficient**  **(95% CI)** | **Standardised coefficient** | **P** |
| **Demographics** | Age | -0.15  (-0.84, 0.55) | -0.06 | 0.68 |  |  |  |
|  | Gender (male) | 4.8  (-9.2, 18.7) | 0.10 | 0.50 |  |  |  |
|  | Current smoker | -1.18  (-15.2, 12.9) | -0.03 | 0.87 |  |  |  |
|  | Smoking pack years | 0.07  (-0.21, 0.36) | 0.08 | 0.61 |  |  |  |
| **Lung function** | FEV_1_ (%pred) | 0.17  (-0.10, 0.44) | 0.18 | 0.21 |  |  |  |
|  | K_CO_ (%pred) | 0.17  (-0.15, 0.49) | 0.16 | 0.29 |  |  |  |
|  | RV/TLC (%) | -0.43  (-1.04, 0.18) | -0.21 | 0.16 |  |  |  |
| **Muscle parameters** | **BMI** | **1.53**  **(0.62, 2.45)** | **0.44** | **0.002** | **1.48**  **(-0.25, 3,20)** | **0.43** | **0.09** |
|  | **FFMI** | **3.01**  **(0.80, 5.23)** | **0.37** | **0.009** | **-0.19**  **(-4.37, 3.98)** | **-0.02** | **0.93** |
|  | QMVC/ BMI | -0.34  (-15.5, 14.8) | -0.01 | 0.96 |  |  |  |
|  | RF_CSA_ | 0.00  (-0.05, 0.06) | 0.02 | 0.91 |  |  |  |
| **Dyspnoea/ Health status** | MMRC | 1.56  (-4.56, 7.68) | 0.07 | 0.61 |  |  |  |
|  | SGRQ | 0.09  (-0.21, 0.40) | 0.09 | 0.54 |  |  |  |
| **Exercise capacity/ Physical activity** | 6MW | 0.01  (-0.06, 0.08) | 0.04 | 0.76 |  |  |  |
|  | Steps/day | 0.00  (-0.00, 0.00) | -0.05 | 0.76 |  |  |  |
| **Serum** | **GDF-15** | **0.05**  **(0.00, 0.01)** | **0.31** | **0.03** | **0.003**  **(-0.001, 0.008)** | **0.19** | **0.17** |

**TABLE E2 Primers used in this study**

| Target | Forward Primer | Reverse Primer |
| --- | --- | --- |
| Mus MyHC2A | CAGCTTGTTGACCTGGGACT | TTGGTGGATAAACTCCAGGC |
| Mus MyHC2B | GTTTGTCCACCAAGTCCTGC | TAGGGTGAGGGAGCTTGAAA |
| Mus MyHC2X | AGCTTGTTGACCTGGGACTC | ACCTTGTGGACAAACTGCAA |
| Mus MyHC1 | TGTGATAGCCTTCTTGGCCT | AGCAGGAGCTGATTGAGACC |
| Mus GDF-15 | GGCTGCATGCCAACCAGAG | TCTCACCTCTGGACTGAGTATTCC |
| Mus atrogin-1 | TCAGCCTCTGCATGATGTTC | TGGGTGTATCGGATGGAGAC |
| Mus CTGF | TGGCGAGATCATGAAAAAGA | AGATGTCATTGTCCCCAGGA |
| Mus MuRF | CGGGCAACGACCGAGTGCAGACGATC | CCAGGATGGCGTAGAGGGTGTCAAAC |
| mus PAI-1 | GACACCCTCAGCATGTTCATC | AGGGTTGCACTAAACATGTCAG |
| Mus RPLPO | GGACCCGAGAAGACCTCCTT | TGCTGCCGTTGTCAAACACC |
| Mus Cyr61 | GGATGAATGGTGCCTTGC | GTCCACATCAGCCCCTTG |
| Hum RPLPO | TCTACAACCCTGAAGTGCTTGATATC | GCAGACAGACACTGGCAACATT |
| Hum GDF-15 | TGCCCGCCAGCTACAATC | TCTTTGGCTAACAAGTCATCATAGGT |
| Hum myostatin | ACATGAACCCAGGCACTGGT | GGTTGTTTGAGCCAATTTTGC |

**FIGURE LEGENDS**

**Figure E1:** ***Rectus femoris* cross-sectional area (RF_CSA_) is suppressed in COPD patients compared to controls**

RF_CSA_ measurements in healthy controls (n=8) and COPD patients from both the RBH (n=44) and SGH (n=44) cohorts. The data are presented in box and whiskers plots displaying the median, IQR, whiskers are to the 90^th^ percentile and outliers are shown.

**Figure E2:** **RF_CSA_ in associated with QMVC in all participants**

RF­_CSA_ and QMVC measured as described in Methods showed a strong positive correlation in all participants in the study (r=0.66, p<0.001).

**Figure E3: Serum 8-oxo-DG is not difference between COPD patients and controls**

Serum 8-oxo-DG was measured in healthy controls (n=25), and COPD patients (n=50).

**Figure E4:** **X-Y plots demonstrating the relationship between GDF-15 mRNA and myostatin mRNA levels when normalised to RPLPO**

GDF-15 and myostatin were determined as described in Methods in patients from the RBH (n=49) cohort and controls (n=21). GDF-15 expression was highly correlated with myostatin expression (r=0.849, p<0.001)

**Figure E5: GDF-15 expression does not affect the number of centralised nuclei.**

Left and right TA muscles from mice (n=8) over-expressing GDF-15 in the right TA were mounted onto cork and stained with haemotoxylin and eosin. The number of fibres and number of centralised nuclei were determined by counting all fibres and nuclei in 4 fields taken at random from level 8 in the muscle. A minimum of 400 fibres were analysed (average =483) for each sample.

**Figure E6: TGF-β but not GDF-15 increases luciferase activity in C2C12 cells**

Cells were grown and transfected as described in Methods before being treated with GDF-15 at 0, 1, 10 or 50mg/mL or 10ng/mL TGF-β for 16h. Firefly luciferase activity was normalised to renilla luciferase activity. Data presented are mean ± SD. Experiments were performed in triplicate and repeated 3 times.
